# Non-Fasting Glucose Measures and Their Clinical Significance in Diabetes Diagnosis and Cardiovascular and Cancer Risk Prediction: A Narrative Review

**DOI:** 10.3390/ijms27114734

**Published:** 2026-05-25

**Authors:** Yutang Wang, David Song, Tongzhi Wu, Eman M. Othman

**Affiliations:** 1 Discipline of Life Science, Institute of Innovation, Science and Sustainability, Federation University Australia, Ballarat, VIC 3350, Australia; 2 School of Medicine, Deakin University, Geelong, VIC 3220, Australia; 3 School of Medicine, College of Health, Adelaide University, Adelaide, SA 5000, Australia; tongzhi.wu@adelaide.edu.au; 4 Department of Biochemistry, Faculty of Pharmacy, Minia University, Minia 61519, Egypt; eman.sholkamy@uni-wuerzburg.de; 5 Department of Biochemistry-I, Biocenter, University of Wuerzburg, 97074 Wuerzburg, Germany

**Keywords:** diabetes, type 2 diabetes, prediabetes, postprandial, nonfasting, diagnosis, mortality, lipid, fatty acid

## Abstract

Several lipid-management guidelines now favor non-fasting lipid measurements for cardiovascular risk assessment. In parallel, this review evaluated the potential clinical utility of non-fasting glucose measures, which may better reflect real-world glycemic responses, capture postprandial dysregulation not detected by fasting glucose, and offer greater practicality in routine clinical settings. Postprandial plasma glucose measured 4–7.9 h after a meal (PPG_4–7.9h_) shows relative stability within this window and appears to be a promising marker for diagnosing diabetes and predicting mortality from cardiovascular disease (CVD) and cancer. Similarly, 2 h plasma glucose during an oral glucose tolerance test performed 4–7.9 h after a meal (2 h PG_OGTT4–7.9h_) demonstrates diagnostic and prognostic value, particularly for diabetes and cardiovascular mortality. Notably, the diagnostic and predictive performance of these non-fasting measures is not inferior to that of traditional fasting glucose assessments. Mechanistically, postprandial hyperglycemia may contribute to CVD through increased oxidative stress and inflammation, endothelial dysfunction, and promotion of atherogenesis and thrombogenesis. It may also increase cancer risk via oxidative stress, inflammation, and insulin-mediated cellular proliferation. In addition, it may enhance lipogenesis to form membrane lipids supporting tumor growth. Further research is required to establish the clinical applicability, optimal thresholds, and generalizability of these non-fasting glucose measures.

## 1. Introduction and Rationale of the Review

Type 2 diabetes mellitus (T2DM) is a complex, chronic metabolic disorder characterized by persistent hyperglycemia resulting from a combination of insulin resistance and relative insulin deficiency [1,2]. T2DM accounts for approximately 90–95% of all diabetes cases worldwide and represents a substantial and growing global health burden [3,4,5]. The global prevalence of T2DM has increased markedly over recent decades, driven by population aging, urbanization, sedentary lifestyle, and rising rates of overweight and obesity [5,6,7].

Chronic hyperglycemia in T2DM is associated with long-term microvascular complications, including retinopathy, nephropathy, and neuropathy, as well as macrovascular complications such as coronary heart disease, stroke, and peripheral arterial disease [8,9,10,11,12,13,14]. In addition, T2DM is associated with an increased risk of cancer incidence [15,16,17] and cancer-related mortality [18,19,20,21].

Globally, an estimated 240 million individuals (44.7%) with diabetes remained undiagnosed in 2021 [22]. Current diagnostic criteria predominantly rely on measurements of glycated hemoglobin (HbA_1c_), fasting plasma glucose, or 2 h plasma glucose following an oral glucose tolerance test (OGTT). Recently, 1 h plasma glucose following an OGTT after fasting has been recognized as a diagnostic criterion [23]. However, these approaches have some limitations. HbA_1c_ lacks sensitivity for detecting acute hyperglycemia in newly diagnosed diabetes [24] and identifies only a minority of total diabetes cases (approximately 30%) [25]. In addition, fasting plasma glucose testing and OGTT require fasting, which may be inconvenient and potentially hazardous for vulnerable individuals, particularly those at risk of hypoglycemia while awaiting blood collection [26].

In contrast, several lipid-management guidelines now recommend non-fasting lipid measurements over fasting tests for cardiovascular risk assessment [26,27,28,29]. These non-fasting assessments include total cholesterol, high-density lipoprotein (HDL) cholesterol, low-density lipoprotein (LDL) cholesterol, and triglycerides. This paradigm shift reflects evidence that non-fasting tests are more convenient and acceptable to patients and are comparable or superior to fasting measurements in predicting cardiovascular disease (CVD) risk [26] and mortality [30].

The shift toward non-fasting lipid testing in cardiovascular risk assessment provides a strong precedent for evaluating non-fasting glucose measures, as both aim to reflect metabolic processes under real-world conditions, where individuals spend the majority of their time in a postprandial state rather than in the relatively brief fasting period [31]. Just as non-fasting lipids capture postprandial dyslipidemia and improve clinical practicality without compromising predictive value, non-fasting glucose measurements capture postprandial glycemic excursions that contribute to oxidative stress, inflammation, and atherogenesis [32,33]. Therefore, incorporating non-fasting glucose metrics may complement existing cardiovascular risk assessment strategies by providing additional insight into cardiometabolic risk while enhancing clinical convenience and feasibility [34,35].

In line with this shift, it may be clinically valuable to explore the potential utility of non-fasting postprandial plasma glucose (PPG), defined as glucose measured within <8 h of fasting according to the American Diabetes Association [3,4]. This review aims to provide an overview of the potential clinical utility of non-fasting glucose in diabetes diagnosis and risk prediction. We first describe temporal patterns of non-fasting PPG and then focus on emerging evidence highlighting the clinical significance of the postprandial window 4–7.9 h after a meal. During this interval, PPG (PPG_4–7.9h_) and 2 h plasma glucose measured during OGTT (2 h PG_OGTT4–7.9h_) appear to have relevance for diabetes diagnosis and for predicting mortality from CVD and cancer. Finally, we examine the molecular mechanisms through which postprandial hyperglycemia may contribute to increased cardiovascular and cancer risk.

## 2. Plasma Glucose with a Postprandial Time of Less than 4 h

### 2.1. Plasma Glucose Returns to Baseline Within 4 h After a Meal

Postprandial glucose excursions reflect the balance between the rate of exogenous glucose appearance from ingested nutrients and the efficiency of glucose disposal by metabolically active tissues, primarily skeletal muscle and adipose tissue [36,37]. The shape of the postprandial glucose curve therefore represents the dynamic interplay between intestinal glucose absorption, insulin secretion, peripheral insulin sensitivity, and suppression of endogenous glucose production over time [38]. In the early postprandial phase, glucose levels are largely determined by the rate of gastric emptying and intestinal carbohydrate absorption [39,40].

**Figure 1:**
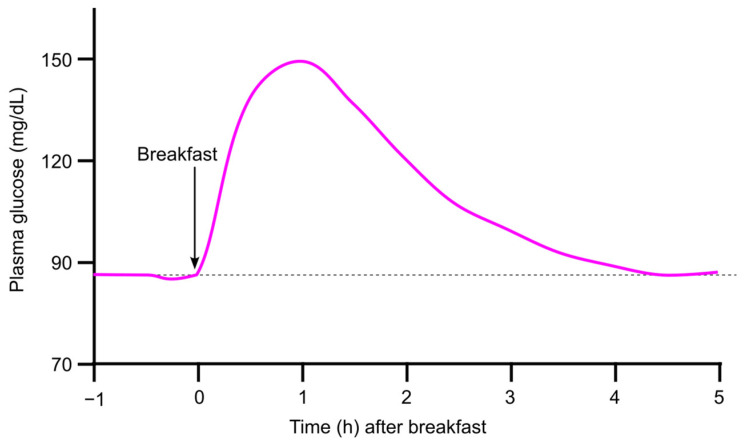
Postprandial plasma glucose (PPG) response in healthy individuals. Fifteen healthy participants consumed a standardized breakfast beginning at 10:00 AM, which was ingested within 5 min. The meal consisted of a cheese omelet and a dextrose-containing beverage, providing an average of 84 g glucose, 10 g fat, and 26 g protein. The data was derived from [41].

In healthy individuals, PPG rises following meal ingestion, typically peaking within 0.5–1 h, and subsequently declines over the next 2–3 h [3,41]. PPG levels generally return to baseline approximately 4 h post-meal (Figure 1) [41].

**Figure 2:**
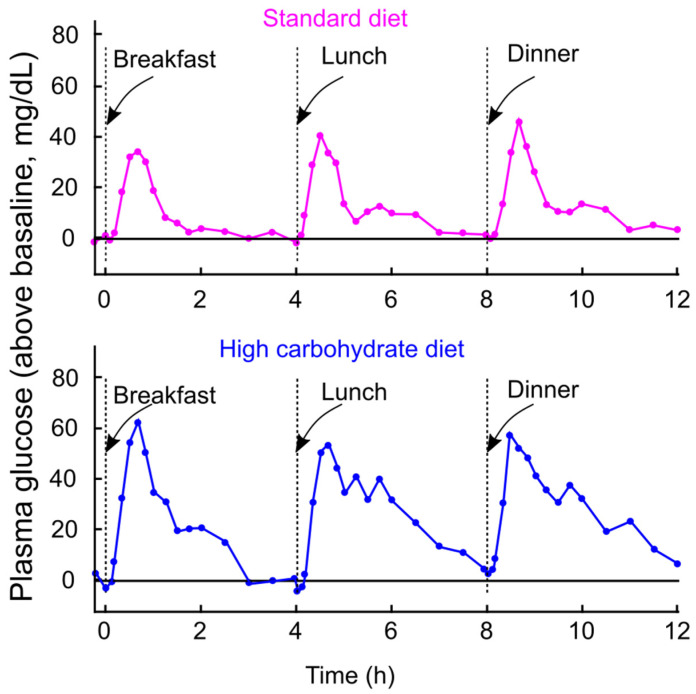
Postprandial glucose responses in healthy individuals following three identical meals. Participants consumed the same standardized meal for breakfast, lunch, and dinner, with meals separated by 4 h. (**Upper**) panel: Twelve participants consumed a standard diet with a macronutrient composition of 40% carbohydrate, 49% fat, and 11% protein. (**Lower**) panel: Ten participants consumed a high-carbohydrate diet with a macronutrient composition of 63% carbohydrate, 27% fat, and 10% protein. The figure is modified from [42], which was published under the terms of the Creative Commons CC BY 4.0 DEED (https://creativecommons.org/licenses/by/4.0/, accessed on 1 March 2026).

Eichenlaub et al. [42] examined PPG dynamics in healthy individuals following consumption of three identical meals. Participants consumed either a standard diet or a high-carbohydrate diet (Figure 2). PPG levels returned to baseline within 3 h after the standard diet, whereas normalization occurred within 4 h following the high-carbohydrate diet. These findings suggest that plasma glucose generally reaches a homeostatic state approximately 4 h after meal intake in healthy individuals, regardless of meal timing or macronutrient composition. Supporting this concept, a large population-based study of 34,907 US adults from the general population demonstrated that PPG attained a relatively stable level 4 h post-meal [43].

### 2.2. PPG Within 4 h After a Meal for Risk Prediction

Non-fasting plasma glucose has been proposed as a marker for risk prediction [44]. Several studies have reported that elevated PPG levels measured 1 or 2 h after breakfast [45,46,47,48] or 2 h after lunch [49] are associated with an increased risk of all-cause mortality. Similarly, higher PPG levels at 1 or 2 h after breakfast [45,46] or 2 h after lunch [49,50] have been linked to an elevated risk of CVD events.

Despite these associations, PPG measurements obtained 1 or 2 h after a meal have limited practicality for large-scale screening and routine clinical use. First, dietary variation in meal composition and quantity can alter PPG levels by more than 20 mg/dL, introducing substantial measurement variability [42]. Second, accurate timing of blood sampling at exactly 1 or 2 h after a meal is difficult to achieve in real-world settings, and even modest deviations (e.g., ±0.5 h [48]) may lead to clinically meaningful differences, given the rapid fluctuations in glucose concentration during the early postprandial period [42]. Third, inter-individual differences in digestion and metabolism, combined with the absence of a universally accepted meal protocol for routine testing [51], further limit the interpretability and utility of PPG measured at 1 or 2 h post-meal. Consistent with these limitations, PPG measured at variable times within 0–3.9 h after a meal was not associated with cardiovascular or cancer mortality in US adults from the general population [43,52].

## 3. PPG Levels Are Stable Between 4 and 7.9 h After a Meal

Postprandial glucose concentrations 4 h after meal ingestion are increasingly influenced by peripheral insulin sensitivity and hepatic glucose output, including both gluconeogenesis and glycogenolysis. Although in most individuals, intestinal glucose absorption is largely complete within 4 h, delayed gastric emptying can prolong carbohydrate delivery to the intestine and extend the contribution of exogenous glucose to circulating levels in a subset of individuals [39,53].

**Figure 3:**
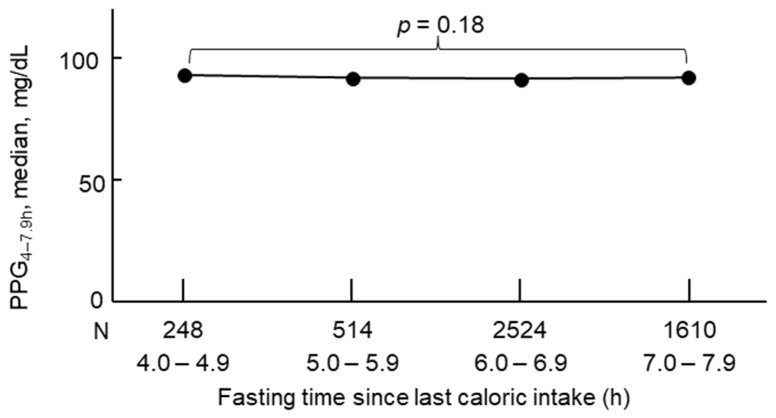
Hourly average of PPG_4–7.9h_. PPG_4–7.9h_, postprandial plasma glucose measured 4–7.9 h after a meal. The figure is from [55], which was published under the terms of the Creative Commons CC BY 4.0 DEED (https://creativecommons.org/licenses/by/4.0/, accessed on 1 March 2026).

Postprandial plasma glucose levels measured between 4 and 7.9 h (PPG_4–7.9h_) exhibit relative stability during this interval (Figure 3) [52,54,55]. Consequently, this late postprandial, non-fasting time window may offer a distinct and practical opportunity for glucose assessment and cardiometabolic risk stratification.

## 4. Clinical Significance of PPG_4–7.9h_

### 4.1. PPG_4–7.9h_ for Diabetes Diagnosis

**Figure 4:**
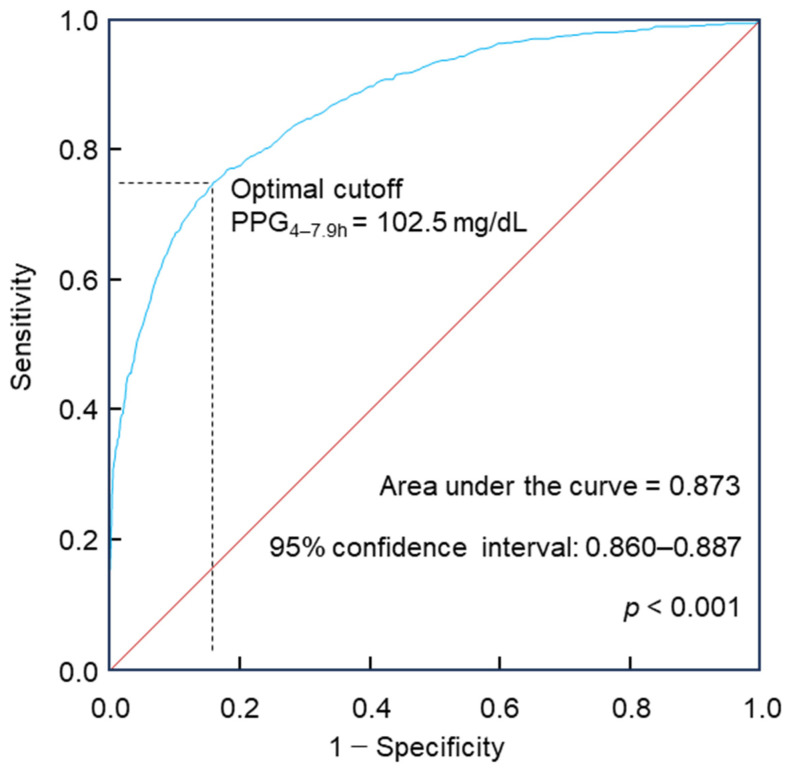
The predictive value of estimated PPG_4–7.9h_ for diabetes. PPG_4–7.9h_, postprandial plasma glucose measured 4–7.9 h after meal consumption. The figure is from [54], which was published under the terms of the Creative Commons CC BY 4.0 DEED (https://creativecommons.org/licenses/by/4.0/, accessed on 1 March 2026).

The diagnostic utility of PPG_4–7.9h_ remains to be fully established. A recent study evaluated whether estimated PPG_4–7.9h_ could be used for diabetes diagnosis [54]. In that study, two participant groups were analyzed. Group 1 (*n* = 4420) had measured PPG_4–7.9h_ values, whereas Group 2 (*n* = 8422) lacked PPG_4–7.9h_ measurements but had complete data for traditional diabetes diagnostic tests [54]. Using data from Group 1, a multiple linear regression model was developed to estimate PPG_4–7.9h_, achieving an accuracy within 11 mg/dL in 80% of participants. This model was subsequently applied to Group 2 to generate estimated PPG_4–7.9h_ values for each individual. Receiver operating characteristic analysis demonstrated that estimated PPG_4–7.9h_ identified diabetes with an overall accuracy of 87.3%, a sensitivity of 75.1%, a specificity of 84.1%, and an optimal cutoff value of 102.5 mg/dL (Figure 4) [54]. This performance falls within the “excellent” diagnostic accuracy range of 80–90% [56,57].

By comparison, the diagnostic accuracy of HbA_1c_ for diabetes has been reported to be approximately 65% [58], and an HbA_1c_ threshold ≥ 6.5% yields sensitivities ranging from 35% to 50% [59,60]. Similarly, a fasting plasma glucose threshold ≥ 126 mg/dL detects OGTT-diagnosed diabetes with sensitivities between 40% and 70% [61,62,63]. Importantly, PPG_4–7.9h_ is a non-fasting measure, offering greater convenience than fasting tests, and its measurement is less costly than HbA_1c_ testing [64,65]. Collectively, these findings suggest that establishing PPG_4–7.9h_ as an additional diagnostic marker for diabetes may have meaningful clinical relevance [54].

Notably, the optimal cutoff value of 102.5 mg/dL for estimated PPG_4–7.9h_ is substantially lower than the current fasting plasma glucose threshold of 126 mg/dL used for diabetes diagnosis [3,66]. This finding is consistent with prior reports. For example, Peter et al. [67] observed mean PPG_4–7.9h_ (measured 4 h post-meal) and fasting plasma glucose concentrations of 102 mg/dL and 127 mg/dL, respectively, in individuals with mild type 2 diabetes. Similarly, Avignon et al. [68] reported corresponding values of 104 mg/dL for PPG_4–7.9h_ and 133 mg/dL for fasting plasma glucose in patients with mild type 2 diabetes, with PPG_4–7.9h_ assessed 5 h after lunch. Collectively, these data indicate that the proposed PPG_4–7.9h_ cutoff of 102.5 mg/dL aligns closely with the reported range of 102–104 mg/dL observed in individuals with early-stage type 2 diabetes [67,68].

The physiological basis for the higher fasting plasma glucose levels relative to PPG_4–7.9h_ remains incompletely understood. However, this difference may be partially attributable to the “dawn phenomenon” [69], characterized by a transient early-morning rise in glucose levels driven by increased hepatic glycogenolysis and gluconeogenesis in individuals with diabetes [70].

### 4.2. PPG_4–7.9h_ for Mortality Risk Prediction from CVD and Diabetes

**Figure 5:**
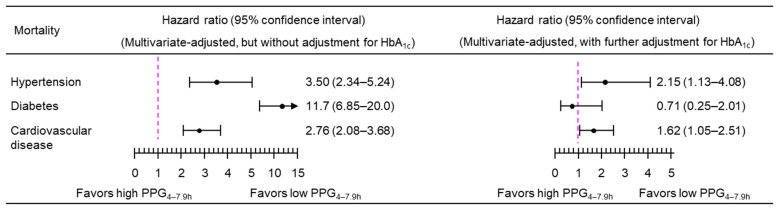
A one-natural-log-unit increase in PPG_4–7.9h_-associated mortality risk in 4896 participants. HbA_1c_, hemoglobin A_1c_; PPG_4–7.9h_, postprandial plasma glucose measured 4–7.9 h after a meal. The image is from [55], which was published under the terms of the Creative Commons CC BY 4.0 DEED (https://creativecommons.org/licenses/by/4.0/, accessed on 1 March 2026).

The prognostic value of PPG_4–7.9h_ for mortality risk was evaluated in 4896 US adults [55]. Higher PPG_4–7.9h_ levels were positively associated with mortality from hypertension, diabetes, and CVD (Figure 5). Importantly, the associations with hypertension- and CVD-related mortality remained significant after adjustment for HbA_1c_. In contrast, the association between PPG_4–7.9h_ and diabetes-related mortality was attenuated after adjustment for HbA_1c_ (Figure 5).

Notably, similar associations were observed among participants without a prior diagnosis of myocardial infarction or stroke, supporting the hypothesis that elevated PPG_4–7.9h_ may contribute to the development of incident CVD rather than merely reflecting established pathology.

### 4.3. PPG_4–7.9h_ for Mortality Risk Prediction from Cancer

The prognostic value of PPG_4–7.9h_ for cancer mortality was evaluated in 4648 US adults from the general population [52]. Higher PPG_4–7.9h_ levels were significantly associated with an increased multivariable-adjusted risk of cancer mortality, with a 1-natural-log-unit increase corresponding to a hazard ratio of 2.00 (95% confidence interval, 1.09–3.67). In contrast, fasting plasma glucose, PPG measured within 0–3.9 h after a meal, HbA_1c_, and insulin were not associated with cancer mortality in this cohort [52]. Notably, the association between PPG_4–7.9h_ and cancer mortality remained robust after additional adjustment for HbA_1c_, indicating superior predictive capacity of PPG_4–7.9h_ for cancer mortality.

The same study also identified PPG_4–7.9h_ thresholds for cancer risk stratification according to HbA_1c_ categories (<5.7%, 5.7–6.4%, ≥6.5%). The corresponding PPG_4–7.9h_ categories were defined as <94 mg/dL (normal), 94–100 mg/dL (borderline high), and ≥101 mg/dL (high) [52]. Compared with individuals with normal PPG_4–7.9h_, those with high PPG_4–7.9h_ (≥101 mg/dL) exhibited a 41% higher risk of cancer mortality [52].

Moreover, elevated PPG_4–7.9h_ appeared to be related to the development of new cancers. Among participants without a prior cancer diagnosis, high PPG_4–7.9h_ was associated with a 45% higher risk of cancer mortality relative to normal PPG_4–7.9h_. In contrast, among the 413 participants with a prior cancer diagnosis, PPG_4–7.9h_ was associated with a non-significant increase in cancer mortality (high vs. normal PPG_4–7.9h_: hazard ratio, 1.64; 95% confidence interval, 0.88–3.07; *p* = 0.12) [52], likely reflecting limited statistical power due to small sample size.

### 4.4. Advantages and Limitations of PPG_4–7.9h_ in Comparison with Established Diabetes Biomarkers

Current diabetes biomarkers—including fasting plasma glucose, HbA_1c_, and OGTT—each have distinct advantages and limitations in terms of sensitivity, reproducibility, and clinical practicality, while the emerging non-fasting glucose measure PPG_4–7.9h_ may provide complementary insights into postprandial dysregulation and cardiometabolic risk [33,43,52,54,55,71,72,73,74,75] (Table 1). Notably, the PPG_4–7.9h_ test does not require the consumption of a standardized test meal; instead, individuals consume their habitual meals. This characteristic may enhance the clinical utility, convenience, and practicality of PPG_4–7.9h_.

## 5. Two-Hour Plasma Glucose During OGTT Performed Between 4 and 7.9 h After a Meal (2 h PG_OGTT@4–7.9h_)

### 5.1. 2 h PG_OGTT@4–7.9h_ for Diabetes Diagnosis

**Figure 6:**
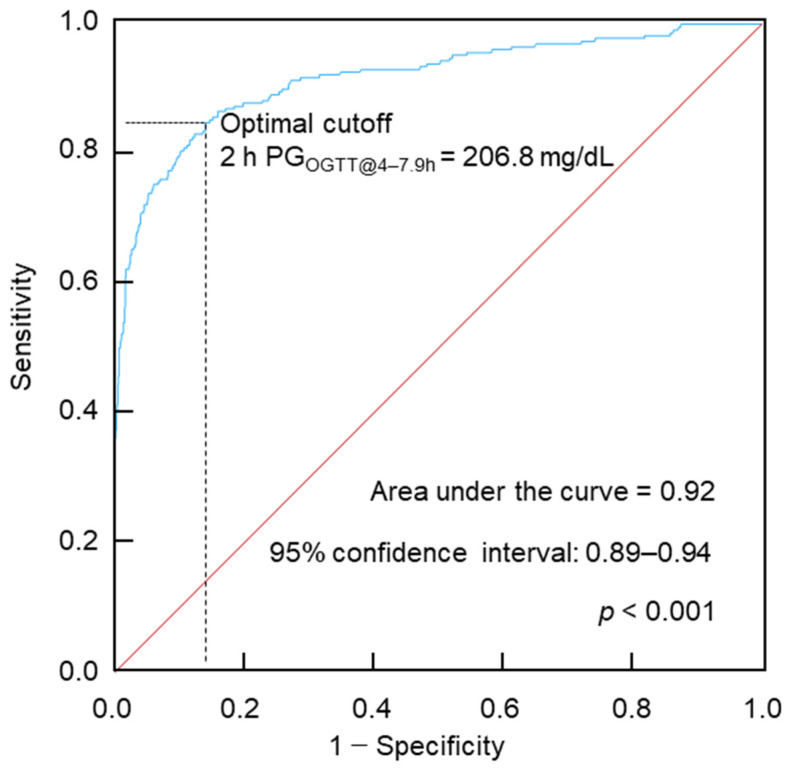
The predictive value of 2 h PG_OGTT@4–7.9h_ for diabetes (defined as hemoglobin A_1c_ ≥ 6.5%). The sensitivity was 84.8%, and the specificity was 86.1%, with an optimal cutoff of 206.8 mg/dL; 2 h PG_OGTT@4–7.9h_, 2 h plasma glucose during an OGTT performed between 4 and 7.9 h after meal consumption. The image is from [76], which was published under the terms of the Creative Commons CC BY 4.0 DEED (https://creativecommons.org/licenses/by/4.0/, accessed on 19 January 2025).

An OGTT is typically performed after at least 8 h of fasting. A recent study examined the potential clinical utility of 2 h plasma glucose during an OGTT performed 4–7.9 h postprandially (termed 2 h PG_OGTT@4–7.9h_), in a cohort of 2347 US adults [76]. In that study, 2 h PG_OGTT@4–7.9h_ exhibited a linear association with HbA_1c_ and was positively associated with an increased adjusted risk of HbA_1c_-defined diabetes [76]. Receiver operating characteristic curve analysis demonstrated that 2 h PG_OGTT@4–7.9h_ predicted HbA_1c_-diagnosed diabetes with an accuracy of 92% and an optimal cutoff value of 206.8 mg/dL (Figure 6), comparable to the performance of fasting 2 h OGTT glucose (accuracy = 95%, optimal cutoff = 203.6 mg/dL) [76]. Notably, diabetes was defined solely by HbA_1c_ in this analysis. Therefore, further studies incorporating comprehensive diagnostic criteria are warranted to determine whether 2 h PG_OGTT@4–7.9h_ can be reliably used for diabetes diagnosis.

### 5.2. 2 h PG_OGTT@4–7.9h_ for Prediabetes Risk

Based on the American Diabetes Association Criteria, individuals with an HbA_1c_ level between 5.7% and 6.4% can be defined as prediabetes. A one-square-root increase in 2 h PG_OGTT@4–7.9h_ was associated with a 13% higher adjusted risk of prediabetes defined by HbA_1c_ [76]. When analyzed as a dichotomous variable using a cutoff of 140 mg/dL, individuals with 2 h PG_OGTT@4–7.9h_ ≥ 140 mg/dL had a 29% higher adjusted risk of prediabetes compared with those with values < 140 mg/dL [76]. Collectively, these findings suggest that 2 h PG_OGTT@4–7.9h_ may be a useful marker for identifying individuals at elevated risk of prediabetes.

### 5.3. 2 h PG_OGTT@4–7.9h_ for Mortality Risk Prediction

A one-square-root increase in 2 h PG_OGTT@4–7.9h_ was associated with a 6%, 46%, and 7% higher risk of mortality from all causes, diabetes, and CVD, respectively (Table 2) [76]. The associations between 2 h PG_OGTT@4–7.9h_ and both all-cause and diabetes-related mortality remained significant after adjustment for HbA_1c_. In contrast, the association with CVD mortality was attenuated after adjustment for HbA_1c_. No significant association was observed between 2 h PG_OGTT@4–7.9h_ and cancer mortality (Table 2).

Notably, the predictive performance of 2 h PG_OGTT@4–7.9h_ for CVD mortality was comparable to that of fasting 2 h OGTT glucose (Table 2). Moreover, 2 h PG_OGTT@4–7.9h_ appeared to be a stronger predictor of diabetes-related mortality than its fasting counterpart (hazard ratio, 1.46 vs. 1.29; Table 2) [76], highlighting its potential clinical value as a non-fasting prognostic marker.

## 6. Mechanisms Linking Postprandial Hyperglycemia to Cardiovascular Disease

### 6.1. Postprandial Hyperglycemia Induces Changes in Glucose Metabolism

**Figure 7:**
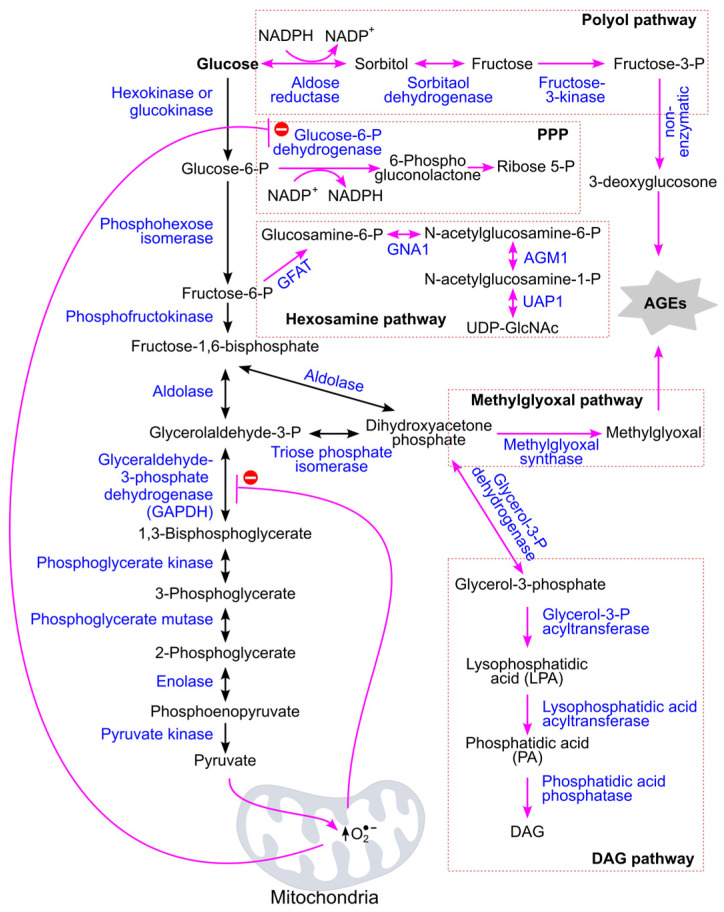
Glucose metabolism and alteration under postprandial hyperglycemia. Under normal conditions, most of the glucose undergoes glycolysis to produce pyruvate (left side of the figure), and the latter then enters mitochondria for the citric acid cycle reaction and ATP production. Under postprandial hyperglycemia, excess superoxide is generated in mitochondria, which then inhibits the enzyme GAPDH, decreasing glycolysis. Consequently, glucose metabolism is diverted to other pathways, including the polyol pathway, the hexosamine pathway, the methylglyoxal pathway, and the DAG pathway. In addition, increasing oxidative stress under the hyperglycemic condition inhibits glucose-6-phosphate dehydrogenase, and thus inhibits the pentose phosphate pathway, leading to decreased production of NADPH. Fuchsia-colored arrows represent altered metabolism pathways due to hyperglycemia. Double-headed arrows indicate reversible reactions. Blue text represents enzymes for each reaction. ↑, increase; AGEs, advanced glycation end-products; AGM1, phosphoacetylglucosamine mutase; DAG, diacyl glycerol; GAPDH, glyceraldehyde-3-phosphate dehydrogenase; GFAT, glutamine:fructose-6-phosphate aminotransferase; GNA1, glucosamine-6-phosphate acetyltransferase; LPA, lysophosphatidic acid; NADP^+^, oxidized nicotinamide adenine dinucleotide phosphate; NADPH, reduced nicotinamide adenine dinucleotide phosphate; P, phosphate; PA, phosphatidic acid; PPP, pentose phosphate pathway; UAP1, UDP-GlcNAc pyrophosphorylase; UDP-GlcNAc, uridine diphosphate *N*-acetylglucosamine. The image is adapted from [95], which was published under the terms of the Creative Commons CC BY 4.0 DEED (https://creativecommons.org/licenses/by/4.0/, accessed on 1 March 2026).

Postprandial hyperglycemia enhances glycolytic flux and induces excessive mitochondrial superoxide production [77]. The resulting increase in reactive oxygen species (ROS) inhibits glyceraldehyde-3-phosphate dehydrogenase (GAPDH), thereby diverting glucose metabolites into alternative pathogenic pathways (Figure 7). These include: (1) the diacylglycerol (DAG) pathway, in which dihydroxyacetone phosphate is metabolized to generate increased levels of DAG, a key second-messenger molecule that activates protein kinase C signaling [78,79,80,81,82,83]; (2) the polyol pathway, in which glucose is reduced to sorbitol at the expense of reduced nicotinamide adenine dinucleotide phosphate (NADPH)—a critical cofactor for glutathione (GSH) recycling—thereby impairing cellular antioxidant defenses [84]. Flux through this pathway also promotes formation of the reactive carbonyl 3-deoxyglucosone [85,86], which contributes to the generation of advanced glycation end-products (AGEs) [87,88]; (3) the methylglyoxal pathway, in which dihydroxyacetone phosphate is converted to methylglyoxal by methylglyoxal synthase [89,90], leading to AGE formation; and (4) the hexosamine biosynthetic pathway, which produces uridine diphosphate *N*-acetylglucosamine (UDP-GlcNAc) [77,91]. UDP-GlcNAc serves as a substrate for protein O-GlcNAcylation, a post-translational modification that modulates cell signaling and gene transcription [92].

In parallel, hyperglycemia suppresses the pentose phosphate pathway (PPP). Hyperglycemia-induced oxidative stress inhibits glucose-6-phosphate dehydrogenase, the rate-limiting enzyme of the PPP [93,94]. As the PPP is a major source of cellular NADPH, its inhibition further compromises redox homeostasis and antioxidant capacity under hyperglycemic conditions (Figure 7).

### 6.2. Mechanisms Underlying Postprandial Hyperglycemia-Induced Increase in CVD Risks

PPG excursions contribute to CVD through mechanisms involving enhanced oxidative stress, inflammation, endothelial dysfunction, accelerated atherogenesis, and pro-thrombotic changes.

#### 6.2.1. Postprandial Hyperglycemia Increases Oxidative Stress

Postprandial hyperglycemia promotes mitochondrial superoxide overproduction, thereby increasing oxidative stress [77,96]. Concurrently, hyperglycemia activates several metabolic pathways that generate reactive carbonyl species. Activation of the polyol pathway and the methylglyoxal pathway results in the overproduction of 3-deoxyglucosone [85,86] and methylglyoxal, respectively, both of which are highly reactive carbonyl compounds. In addition, postprandial hyperglycemia facilitates the formation of glyoxal through glucose auto-oxidation, contributing further to the cellular reactive carbonyl burden [97]. These reactive carbonyls readily modify intracellular and extracellular proteins by reacting with amino groups, leading to the formation of AGEs [87,88]. Subsequent engagement of AGEs with their cognate receptors (RAGEs) amplifies oxidative stress and triggers pro-inflammatory signaling cascades [88].

**Figure 8:**
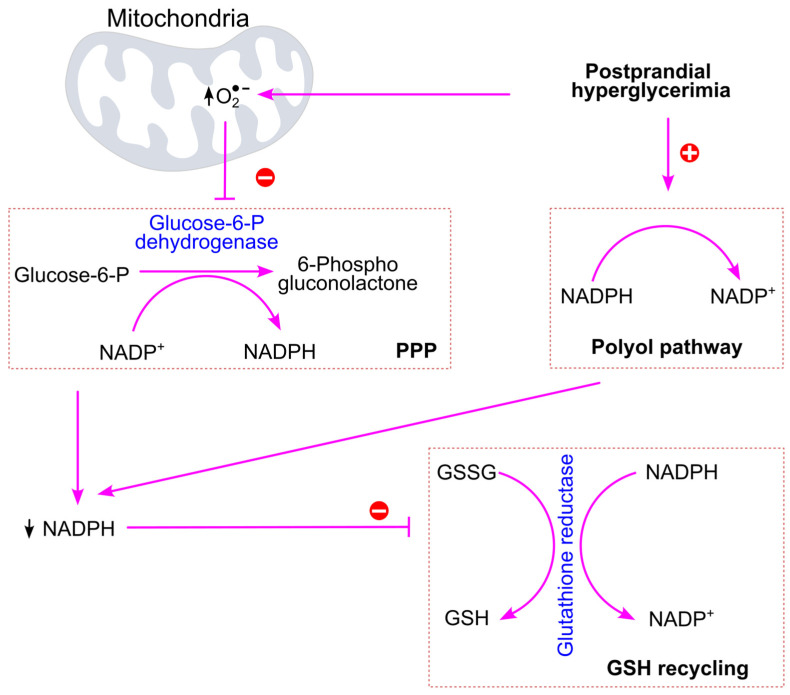
Postprandial hyperglycemia decreases cellular antioxidant capacity. Postprandial hyperglycemia inhibits the pentose phosphate pathway and consequently decreases NADPH production. In addition, it increases the polyol pathway and thus increases NADPH consumption. Therefore, postprandial hyperglycemia leads to NADPH deficiency, consequently decreasing cellular levels of GSH and cellular antioxidant capacity. Blue text represents enzymes for each reaction. ↑, increase; ↓, decrease; GSH, reduced glutathione; GSSG, glutathione disulfide; NADP^+^, oxidized nicotinamide adenine dinucleotide phosphate; NADPH, reduced nicotinamide adenine dinucleotide phosphate; O_2_^●−^, superoxide; P, phosphate; PPP, pentose phosphate pathway.

Postprandial hyperglycemia also disrupts cellular antioxidant defenses by reducing intracellular GSH levels (Figure 8), the cell’s primary endogenous antioxidant. GSH directly neutralizes ROS and reactive nitrogen species (RNS)—including superoxide, hydrogen peroxide, hydroxyl radicals, nitric oxide, and peroxynitrite—via its redox-active thiol group [98,99]. The pentose phosphate pathway (PPP; Figure 7) plays a critical role in maintaining redox homeostasis as the principal source of nicotinamide adenine dinucleotide phosphate (NADPH) [100]. Hyperglycemia suppresses PPP activity, thereby reducing NADPH production [93,94]. Simultaneously, increased flux through the polyol pathway further depletes NADPH due to its high reductive demand. The resulting NADPH deficiency compromises the capacity of glutathione reductase to regenerate reduced GSH from oxidized glutathione (GSSG) [101]. Consequently, intracellular GSH levels decline (Figure 8), leading to impaired antioxidant capacity and redox imbalance [102]. This failure of antioxidant defenses permits excessive accumulation of ROS, such as superoxide and hydrogen peroxide, thereby exacerbating cellular oxidative damage.

#### 6.2.2. Postprandial Hyperglycemia Leads to Activation of Inflammatory Pathways

Postprandial hyperglycemia increases inflammation in humans, as indicated by an increase in high-sensitivity C-reactive protein [103,104]. Hyperglycemia increases superoxide production, which activates nuclear factor-κB (NF-κB) [105], a transcriptional factor, in endothelial and mononuclear cells [106,107], upregulating adhesion molecules such as monocyte chemoattractant protein 1 (MCP-1), vascular cell adhesion protein 1 (VCAM-1), and intercellular adhesion molecule 1 (ICAM-1) [108,109]. In addition, NF-κB increases the transcription of inflammatory mediators [e.g., interleukin-1 (IL-1), IL-6, and tumor necrosis factor-α (TNF-α)] and increases their circulating concentrations [110,111].

Postprandial hyperglycemia activates the DAG–protein kinase C pathway [112,113,114], AGE pathways [115], and the hexosamine pathway [92]. These pathways can activate NF-kB, leading to leukocyte adhesion and inflammation.

#### 6.2.3. Postprandial Hyperglycemia Leads to Endothelial Dysfunction

Spikes of postprandial glucose can induce endothelial dysfunction in both healthy [116,117] and diabetic individuals [118]. The high glucose-induced endothelial dysfunction is due to increased ROS production, as endothelial dysfunction is prevented by ROS scavengers such as GSH [116], superoxide dismutase, and catalase [119,120]. Acute glucose excursion-induced endothelial dysfunction may involve a reduced availability of nitric oxide (NO), as this dysfunction could be alleviated by arginine [121]. Therefore, the underlying mechanism for hyperglycemia-induced endothelial dysfunction is largely derived from an increase in ROS production and a decrease in cellular antioxidant capacity.

#### 6.2.4. Postprandial Hyperglycemia Promotes Atherogenesis

Postprandial hyperglycemia leads to endothelial dysfunction, and the latter contributes to atherosclerosis [78,122]. It also promotes LDL oxidation [116,123,124], resulting from excessive ROS production [118,123]. Oxidized LDL is a major driver of atherosclerosis [125]. Postprandial hyperglycemia increases the production of reactive carbonyl species via the polyol pathway [85,86] and the methylglyoxal pathway [89,90]. The increase in reactive carbonyl species results in the production of AGEs, and the latter bind to RAGEs and amplify oxidative stress and inflammation, thus promoting atherosclerosis [126]. Postprandial hyperglycemia may predict atherosclerosis better than its fasting counterpart or HbA_1c_ [127].

#### 6.2.5. Postprandial Hyperglycemia Enhances Thrombotic Events

Postprandial hyperglycemia leads to a prothrombotic state, which has been reviewed previously [128]. In summary, postprandial hyperglycemia can result in platelet hypersensitivity, enhanced levels of pro-coagulation mediators (e.g., tissue factor and thrombin), and decreased fibrinolysis [128]. Therefore, postprandial hyperglycemia could lead to the development of fibrinolysis-resistant clots and increase the risk of CVD. Postprandial hyperglycemia may be a stronger predictor of thrombotic events than fasting plasma glucose [108].

## 7. Mechanisms Linking Postprandial Hyperglycemia to Cancer

Diabetes is associated with high mortality in patients with various cancers [18,19,20,21]. Cancer incidence is increasing in people with diabetes [15,16,17]. It has been reported that PPG is a better predictor of cancer mortality than fasting glucose or HbA_1c_ [52], highlighting the danger of post-meal spikes. The mechanisms underlying hyperglycemia-associated increase in cancer risk are not well understood, but it may result from an increase in oxidative stress, circulating insulin, and lipogenesis [129].

### 7.1. Postprandial Hyperglycemia Increases ROS Production and Inflammation

**Figure 9:**
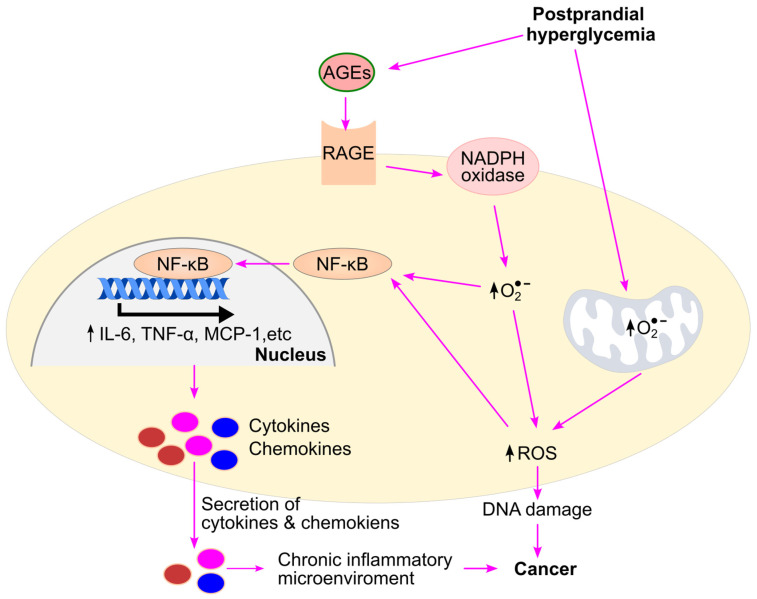
Postprandial hyperglycemia promotes cancer formation via increasing ROS production. ↑, increase; AGE, advanced glycation end-product; IL, interleukin; MCP-1, monocyte chemoattractant protein-1; NADPH, reduced nicotinamide adenine dinucleotide phosphate; NF-κB, nuclear factor-κB; O_2_^●−^, superoxide; RAGE, receptor for advanced glycation end-product; ROS, reactive oxygen species; TNF-α, tumor necrosis factor-alpha.

Postprandial hyperglycemia increases ROS production via mitochondria, which results in DNA damage, contributing to cancer formation [17,130]. Hyperglycemia can increase the formation of AGEs, and the latter increase cancer risk [131]. AGEs bind to RAGEs, leading to activation of NADPH oxidase. Activated NADPH oxidase promotes ROS production [132], causing DNA damage and mutation, and promoting tumorigenesis [133,134]. In addition, activation of NADPH oxidase leads to activation of transcriptional factor NF-κB, which increases gene expression of pro-inflammatory cytokines and chemokines such as IL-6, TNF-α, and MCP-1 [133,135,136], and forms a chronic inflammatory microenvironment to facilitate cancer cell proliferation (Figure 9).

### 7.2. Postprandial Hyperglycemia Promotes Cancer Proliferation Through Increased Circulating Insulin

Postprandial hyperglycemia leads to an increase in circulating insulin, which acts via insulin receptors to directly promote tumor growth [137]. Activation of the insulin receptor leads to the activation of the phosphatidylinositol 3-kinase/protein kinase B/mammalian target of the rapamycin (PI3K/AKT/mTOR) pathway, leading to cancer cell proliferation [138,139,140]. Activation of the insulin receptor can also activate Ras/MAPK (mitogen-activated protein kinase) cascade and promote cancer cell proliferation [141]. Moreover, high insulin can increase the levels of insulin-like growth factor-1, and the latter can also activate Ras/MAPK cascade to promote cancer cell proliferation [142].

### 7.3. Postprandial Hyperglycemia Drives Lipogenesis to Support Cancer Cell Proliferation

**Figure 10:**
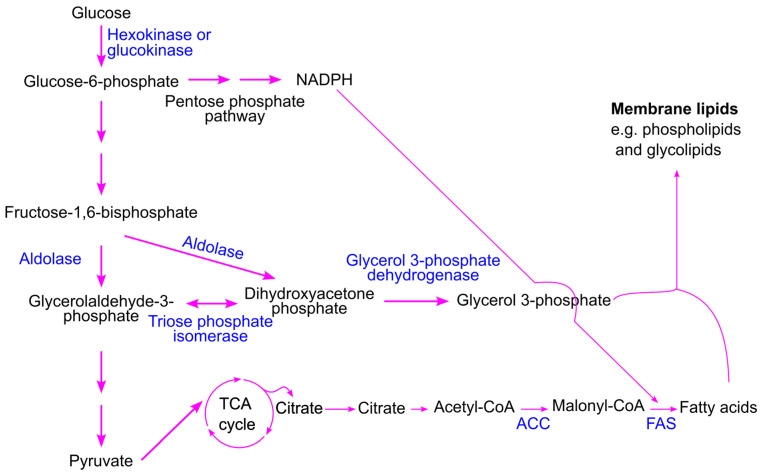
Cellular conversion of excess glucose to membrane lipids. Double-headed arrows indicate reversible reactions. ACC, acetyl-CoA carboxylase; FAS, fatty acid synthase; NADPH, reduced nicotinamide adenine dinucleotide phosphate; TCA, tricarboxylic acid. Blue text represents enzymes. The image is adapted from [95], which was published under the terms of the Creative Commons CC BY 4.0 DEED (https://creativecommons.org/licenses/by/4.0/, accessed on 1 March 2026).

**Figure 11:**
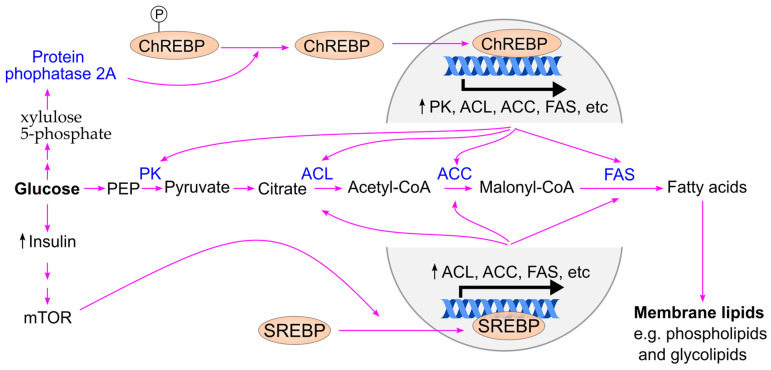
Chronic high postprandial glucose levels promote membrane lipid synthesis via transcriptional regulation. Elevated glucose stimulates the expression of genes involved in lipid synthesis by activating two transcriptional factors: carbohydrate response element-binding protein (ChREBP) and sterol regulatory element-binding protein (SREBP). ↑, increase; ACC, acetyl-CoA carboxylase; ACL, ATP citrate lyase; FAS, fatty acid synthase; mTOR, mammalian target of rapamycin; P, phosphate; PEP, phosphoenopyruvate; PK, pyruvate kinase. Blue text represents enzymes. The image is adapted from [95], which was published under the terms of the Creative Commons CC BY 4.0 DEED (https://creativecommons.org/licenses/by/4.0/, accessed on 1 March 2026).

Excess postprandial glucose is converted into fatty acids and triglycerides [95]. High triglycerides are positively associated with higher risks for diabetes incidence and related mortality [143,144,145] and with higher mortality risks for certain cancers, such as gastric cancer [20]. In addition, increased fatty acid formation promotes the formation of membrane lipids such as phospholipids and glycolipids (Figure 10). Thus, postprandial hyperglycemia may be a significant driver of cancer progression by providing necessary components for cell membrane formation, which are critical for the uncontrolled growth of cancer cells. Postprandial hyperglycemia can enhance lipogenesis via transcriptional regulation, which has been reviewed recently and summarized in Figure 11 [95]. Importantly, hyperglycemia can activate two transcriptional factors—sterol regulatory element-binding protein (SREBP) and carbohydrate response element-binding protein (ChREBP)—to enhance expression of genes involved in fatty acid synthesis, including acetyl-CoA carboxylase, ATP citrate lyase, fatty acid synthase [127,128].

## 8. Limitations and Future Research

More research needs to be conducted to diagnose, prevent, and treat diabetes and diabetes associated complications [146,147,148]. The true diagnostic performance of PPG_4–7.9h_ and 2 h PG_OGTT@4–7.9h_ remains uncertain. To date, the diagnostic accuracy of PPG_4–7.9h_ has been evaluated using estimated rather than directly measured values, while the performance of 2 h PG_OGTT@4–7.9h_ has been examined using HbA_1c_-defined diabetes as the reference standard. Consequently, definitive conclusions regarding their diagnostic validity cannot yet be drawn. Future investigations should employ more rigorous study designs, ideally incorporating cohorts in which each participant undergoes comprehensive assessment with all established diabetes diagnostic tests alongside these two non-fasting glucose measures. In addition, further research is required to determine whether PPG_4–7.9h_ and 2 h PG_OGTT@4–7.9h_ can reliably identify individuals at high risk of prediabetes. If so, future studies should also establish appropriate cutoff values for impaired glucose tolerance and normal glucose tolerance.

Both PPG_4–7.9h_ and 2 h PG_OGTT@4–7.9h_ have demonstrated associations with cardiovascular mortality risk in US adults. However, the generalizability of these findings to other populations remains unknown and this warrants investigation. Moreover, whether these non-fasting glucose measures can predict incident CVD and cancer has yet to be established and should be addressed in future prospective studies.

The traditional 2 h plasma glucose measured during the OGTT has long been used to diagnose impaired glucose tolerance and diabetes. However, growing evidence indicates that 1 h plasma glucose (≥155 mg/dL or 8.6 mmol/L) may be a more sensitive marker of early dysglycemia [149]. Numerous studies demonstrate that an elevated 1 h PG (≥155 mg/dL or 8.6 mmol/L), even among individuals with normal 2 h PG values, is strongly associated with a substantially increased risk of progression to prediabetes, type 2 diabetes, and cardiometabolic disease [23,149,150,151]. In addition, a 1 h plasma glucose value of 209 mg/dL (11.6 mmol/L) has been proposed as a diagnostic threshold for type 2 diabetes [23]. Therefore, it is worthwhile to investigate whether 1 h plasma glucose measured during an OGTT performed between 4 and 7.9 h after a meal (1 h PG_OGTT@4–7.9h_) can be used to diagnose prediabetes and diabetes and to predict cardiometabolic diseases and their complications. In addition, the diagnostic and cardiometabolic risk-predictive performance of PPG_4–7.9h_, 1 h PG_OGTT@4–7.9h_, and 2 h _PGOGTT@4–7.9h_ should be compared with routine fasting 1 h and 2 h plasma glucose measurements obtained during the standard OGTT.

PPG_4–7.9h_ has been reported to be associated with cancer mortality [52]; however, that report did not provide information on specific cancer types. Type 2 diabetes is associated with an increased risk of cancer in general, but this association is heterogeneous across cancer sites [152,153]. Epidemiological studies report higher risks of pancreatic, endometrial, breast, liver, colorectal, gallbladder, and kidney cancers among individuals with type 2 diabetes, and the associations with pancreatic, endometrial, and breast cancers are further supported by Mendelian randomization studies [152,153]. In contrast, type 2 diabetes is not associated with an increased risk of certain cancers (e.g., melanoma and esophageal cancer [153]) and is even associated with a reduced risk of prostate cancer [152,153]. Therefore, future research should investigate the associations between PPG_4–7.9h_ and the incidence and mortality of specific cancer types.

Variability in intestinal glucose absorption [154], differences in gastrointestinal motility—including delayed gastric emptying [39]—and the presence of insulin resistance [155] all contribute to inter-individual variability in postprandial glucose responses. It is therefore important to determine whether these factors influence diagnostic accuracy when PPG_4–7.9h_ is used as a diagnostic criterion for diabetes. In addition, it warrants investigation whether elevations in PPG_4–7.9h_ under these conditions predict an increased incidence of diabetes and a higher risk of cardiometabolic disease.

Liver disease [156], the use of serotonin reuptake inhibitors [157], and corticosteroid therapy [158] are recognized risk factors for the development of type 2 diabetes. In addition, other conditions, such as gastroparesis, as well as medications that affect gastrointestinal motility, may distort glucose levels during the 4–7.9 h postprandial period. Whether elevated PPG_4–7.9h_ induced by these conditions predicts the incidence of type 2 diabetes, as well as cardiovascular and cancer risk, warrants further investigation.

Most of the cited studies are based on U.S. populations; therefore, the clinical significance of PPG_4–7.9h_ and 2 h PG_OGTT4–7.9h_ should be validated in more diverse populations, including different ethnic groups, age ranges, and socioeconomic contexts. Although multiple studies and findings are discussed in this review, it is not a meta-analysis that quantitatively evaluates the consistency and strength of the evidence. Therefore, as more clinical data from diverse populations become available, a formal meta-analysis will be necessary to rigorously assess the accumulated evidence.

## 9. Conclusions

Recent evidence indicates that the postprandial interval of 4–7.9 h may represent a critical window for diabetes diagnosis and for stratifying risk of CVD and cancer. Nevertheless, these observations require confirmation in studies employing more rigorous designs and encompassing diverse populations. Notably, several clinical guidelines have shifted from fasting lipid measurements toward non-fasting testing paradigms [26,27,28,29,159]. In this context, evaluating and validating the clinical utility of non-fasting glucose measures, particularly PPG_4–7.9h_ and 2 h PG_OGTT@4–7.9h_, across diverse populations has substantial translational relevance.

## Figures and Tables

**Table 1:** Advantages and limitations of diabetes biomarkers.

Markers	Advantages	Limitations
FPG	Simple, inexpensive, widely available High specificity for diagnosis	Requires fasting Affected by stress, illness Misses postprandial dysregulation Low sensitivity for early disease
HbA1c	No fasting required Not affected by stress Reflects 2–3 months of glycemic exposure Convenient for screening	Affected by anemia hemoglobinopathies Ethnic variability Relatively more expensive Limited availability Less sensitive for early hyperglycemia
Conventional OGTT (2 h PG)	Detects impaired glucose tolerance (IGT) Captures post-load glucose dynamics	Time-consuming Nausea after ingestion of glucose load Requires fasting Poor reproducibility
PPG_4–7.9h_	No fasting required Convenient Individual takes habitual meals Reflects real-world metabolism Most tests can be performed on the same day as a routine clinical visit Associated with CVD and cancer risk May diagnose diabetes with a high accuracy (87%)	Evidence still emerging Need validation

CVD, cardiovascular disease; FPG, fasting plasma glucose; HbA1c, hemoglobin A1c; OGTT, oral glucose tolerance test; PG, plasma glucose; PPG_4–7.9h_, postprandial plasma glucose measured 4–7.9 h after a meal.

**Table 2:** Association between 2 h plasma glucose during OGTT ^1^ with mortality, with or without adjustment for HbA_1c_.

Mortality	Multivariate Adjusted, Without Adjustment for HbA_1c_			Multivariate Adjusted, with Further Adjustment for HbA_1c_		
	Hazard Ratio	95% CI	*p*	Hazard Ratio	95% CI	*p*
2 h PG_OGTT@4–7.9h_						
All-cause mortality	1.06	1.04–1.08	<0.001	1.04	1.02–1.07	0.003
Diabetes mortality	1.46	1.33–1.61	<0.001	1.39	1.17–1.65	<0.001
CVD mortality	1.07	1.03–1.11	<0.001	1.03	0.98–1.08	0.26
Cancer mortality	1.00	0.95–1.05	1.00	1.02	0.96–1.07	0.58
2 h PG during OGTT after fasting						
All-cause mortality	1.06	1.04–1.07	<0.001	1.04	1.02–1.06	<0.001
Diabetes mortality	1.29	1.21–1.38	<0.001	1.26	1.12–1.43	<0.001
CVD mortality	1.06	1.03–1.09	<0.001	1.01	0.97–1.04	0.77
Cancer mortality	1.00	0.97–1.04	0.80	1.00	0.96–1.05	0.85

^1^, square root transformed; 2 h PG_OGTT@4–7.9h_, 2 h plasma glucose during an OGTT between 4 and 7.9 h after a meal; CI, confidence interval; HbA_1c_, hemoglobin A_1c_; OGTT, oral glucose tolerance test; PG, plasma glucose. The table is from [76], which was published under the terms of the Creative Commons CC BY 4.0 DEED (https://creativecommons.org/licenses/by/4.0/, accessed on 19 January 2025).

## Data Availability

Not applicable.

## Abbreviations

The following abbreviations are used in this manuscript:

2 h PG_OGTT@4–7.9h_	2 h plasma glucose during an OGTT performed between 4 and 7.9 h after a meal
ACC	acetyl-CoA carboxylase
ACL	ATP citrate lyase
AGE	advanced glycation end-product
AGM1	phosphoacetylglucosamine mutase
CI	confidence interval
CVD	cardiovascular disease
DAG	diacyl glycerol
FAS	fatty acid synthase
GSH	reduced glutathione
GSSG	glutathione disulfide
GAPDH	glyceraldehyde-3-phosphate dehydrogenase
GFAT	glutamine:fructose-6-phosphate aminotransferase
GNA1	GlcN-6P acetyltransferase
h	hour
HbA_1c_	hemoglobin _A1c_
HDL	high-density lipoprotein
HR	hazard ratio
ICAM-1	intercellular adhesion molecule 1
IL	interleukin
LDL	low-density lipoprotein
LPA	lysophosphatidic acid
MAPK	mitogen-activated protein kinase
MCP-1	monocyte chemoattractant protein-1
mTOR	mammalian target of rapamycin
NADP^+^	oxidized nicotinamide adenine dinucleotide phosphate
NADPH	reduced nicotinamide adenine dinucleotide phosphate
NF-κB	nuclear factor-κB
O_2_^●−^	superoxide
NO	nitric oxide
OGTT	oral glucose tolerance test
P	phosphate
PA	phosphatidic acid
PEP	phosphoenopyruvate
PK	pyruvate kinase
PPG	postprandial plasma glucose
PPG_4–7.9h_	postprandial plasma glucose measured 4–7.9 h after a meal
PPP	pentose phosphate pathway
RAGE	receptor for advanced glycation end-product
ROS	reactive oxygen species
T2DM	type 2 diabetes mellites
TCA	tricarboxylic acid
TNF-α	tumor necrosis factor-alpha
UAP1	UDP-GlcNAc pyrophosphorylase
UDP-GlcNAc	uridine diphosphate *N*-acetylglucosamine
VCAM-1	vascular cell adhesion protein 1
